# Firing frequency and entrainment maintained in primary auditory neurons in the presence of combined BDNF and NT3

**DOI:** 10.1038/srep28584

**Published:** 2016-06-23

**Authors:** Tess Wright, Lisa N. Gillespie, Stephen J. O’Leary, Karina Needham

**Affiliations:** 1 Department of Surgery (Otolaryngology), University of Melbourne, Royal Victorian Eye & Ear Hospital, East Melbourne, Victoria, Australia; 2 Bionics Institute, East Melbourne, Victoria, Australia; 3 Office for Research Ethics and Integrity, University of Melbourne, Parkville, Victoria, Australia

## Abstract

Primary auditory neurons rely on neurotrophic factors for development and survival. We previously determined that exposure to brain-derived neurotrophic factor (BDNF) and neurotrophin-3 (NT3) alters the activity of hyperpolarization-activated currents (*I*_h_) in this neuronal population. Since potassium channels are sensitive to neurotrophins, and changes in *I*_h_ are often accompanied by a shift in voltage-gated potassium currents (*I*_K_), this study examined *I*_K_ with exposure to both BDNF and NT3 and the impact on firing entrainment during high frequency pulse trains. Whole-cell patch-clamp recordings revealed significant changes in action potential latency and duration, but no change in firing adaptation or total outward *I*_K_. Dendrotoxin-I (DTX-I), targeting voltage-gated potassium channel subunits K_V_1.1 and K_V_1.2, uncovered an increase in the contribution of DTX-I sensitive currents with exposure to neurotrophins. No difference in Phrixotoxin-1 (PaTX-1) sensitive currents, mediated by K_V_4.2 and K_V_4.3 subunits, was observed. Further, no difference was seen in firing entrainment. These results show that combined BDNF and NT3 exposure influences the contribution of K_V_1.1 and K_V_1.2 to the low voltage-activated potassium current (*I*_KL_). Whilst this is accompanied by a shift in spike latency and duration, both firing frequency and entrainment to high frequency pulse trains are preserved.

Neurotrophins are critical in the development and maintenance of neural systems. It is now well established that loss of neurotrophins results in neural degeneration, and exposure to either endogenous or exogenous neurotrophins promotes neuronal survival^1^. Whilst there is growing evidence that neurotrophins can also modulate ion channel activity and expression^2^,^3^,^4^,^5^,^6^,^7^, the details of which channels are affected, and how this influences neural communication is still being resolved.

BDNF and NT3 are two of the key endogenous neurotrophins present in the peripheral auditory system^8^,^9^. Primary auditory neurons (spiral ganglion neurons; SGNs) are reliant on the presence of both BDNF and NT3 for development^10^,^11^,^12^,^13^,^14^, and the expression of BDNF and NT3 is also found in the postnatal cochlea^15^. Loss of neurotrophic support following hair cell death has been linked with degeneration of SGNs in deafness^16^,^17^, and their rescue has been demonstrated with delivery of exogenous neurotrophins^16^,^18^,^19^,^20^,^21^. These findings have highlighted the potential of neurotrophins in treatments of hearing loss^22^.

To determine the functional outcomes of neurotrophin delivery, both *in vivo* and *in vitro* studies have examined the impact of BDNF and/or NT3 on neuronal activity^6^,^23^,^24^,^25^. Initial *in vitro* studies revealed that BDNF and NT3 had opposing effects on SGNs, and suggested that application of one or the other would propagate different firing patterns^2^. Given that the clinical application of neurotrophins is guided by the desire to preserve native firing properties, our earlier investigation examined outcomes with combined BDNF and NT3^6^, and determined that firing adaptation was maintained when both neurotrophins were present. These findings match those seen in the oculomotor system, where BDNF and NT3 applied individually drive either tonic or phasic firing, but combined exposure maintains the endogenous firing sensitivity^26^.

Yet despite the maintenance of firing activity with combined application of BDNF and NT3, our previous investigation also revealed a significant increase in *I*_h_^6^. This current is mediated by the family of hyperpolarisation-activated cyclic nucleotide-gated (HCN) channels and has been implicated in setting resting membrane potential, lowering input resistance and promoting rapid firing through faster temporal integration^27^,^28^. It has also been linked with a change in firing frequency in deafness/sensory deprivation^29^,^30^. However, despite a significant shift in the activity of *I*_h_ with exposure to BDNF and NT3, little difference was observed in the key physiological features (resting membrane potential, input resistance or firing rate) of neurotrophin-treated and untreated neurons^6^. Moreover, measures of voltage sag in current-clamp were consistent between the two groups. This finding suggested that a concurrent increase in outward potassium currents (*I*_K_) may offset the increase in *I*_h_.

A number of studies have identified a correlation between the activity of *I*_h_ and *I*_K_^31^,^32^,^33^,^34^. In both cochlear nucleus and medial superior olive, variations in *I*_h_ are counterbalanced by changes in the contribution of low-voltage-activated potassium currents (*I*_KL_)^31^,^34^. MacLean and colleagues^32^,^33^ also identified a similar co-regulation of *I*_h_ and the transient A-type potassium current (*I*_A_) in rhythmically active neurons. Interestingly, expression of the potassium channel subunits that contribute to *I*_KL_ and *I*_A_ in primary auditory neurons are both subject to modulation by BDNF and NT3^2^. The low-voltage activated *I*_KL_ is linked with expression of potassium channel subunits K_V_1.1, K_V_1.2 and K_V_1.6^35^,^36^,^37^. Alternatively, *I*_A_ is thought to be mediated by K_V_4.2, K_V_4.3 and K_V_3.4 subunits^2^,^35^. Previous studies demonstrated that expression of potassium channel subunit K_V_4.2 increased in the presence of NT3, whilst both K_V_1.1 and K_V_4.2 increased when exposed to exogenous BDNF^2^. Together, these findings suggest that one or both of *I*_A_ and *I*_KL_ could change in concert with *I*_h_ in the presence of both NT3 and BDNF.

This paper examines the contribution of voltage-gated potassium channels K_V_1.1 and K_V_1.2, and K_V_4.2 and K_V_4.3 to *I*_K_ in SGNs with exposure to both BDNF and NT3 *in vitro*. Further, it investigates what influence the neurotrophin-mediated modulation of ion channels has on firing responses to pulse trains.

## Results

Data presented here represent analysis of recordings from 128 SGNs cultured either in media supplemented with both BDNF and NT3 (each at 10 ng/ml; n = 79) or without neurotrophin supplements (n = 49). These populations will be described hereafter as neurotrophin-treated (NT) or untreated (UT) respectively.

### Electrophysiological characteristics

**Figure 1:**
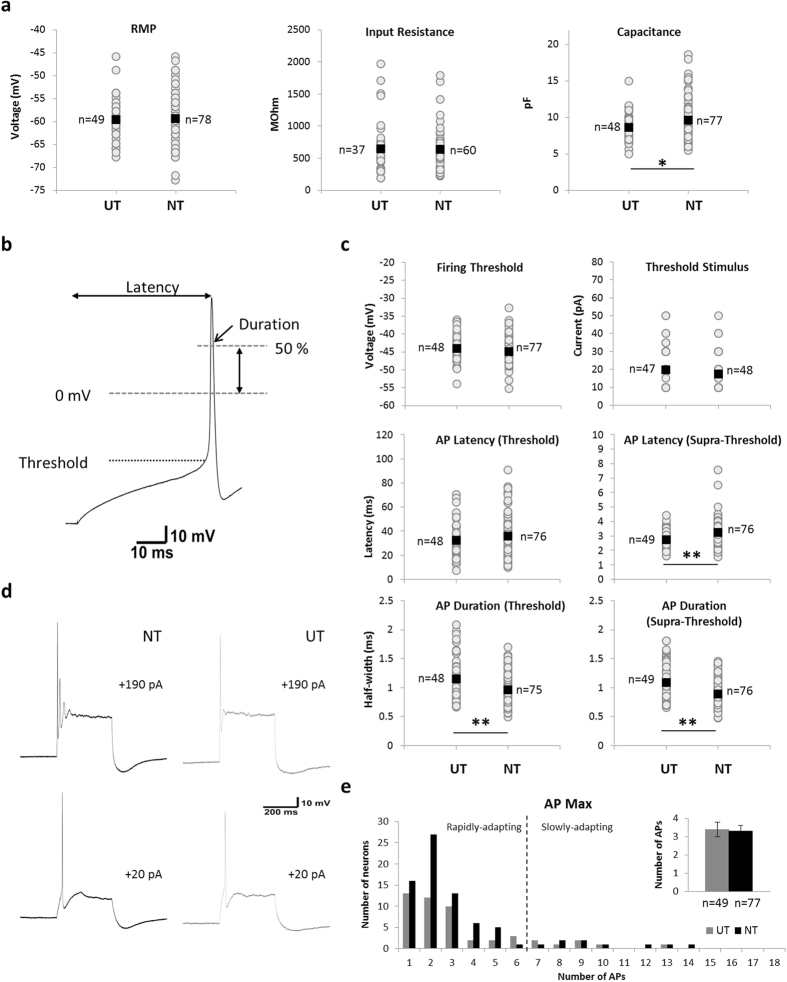
Electrophysiological properties of SGN neurons. (**a)** Comparison of passive membrane properties (resting membrane potential [RMP], input resistance and capacitance) between populations. Responses of individual neurons (gray circles) and sample means (black square) are both shown. (**b)** Typical action potential fired by an SGN in response to membrane depolarization (example shown is an NT neuron). Latency was measured as the time between stimulus onset and the peak of the action potential. Action potential threshold voltage was determined as the voltage at the mid-point of the rising phase, measured at the lowest stimulus current to evoke firing. Action potential duration was measured as the width of the peak at 50% of the overshooting component (i.e. above 0 mV). (**c)** Firing properties of the SGN population. Both individual responses (gray circles) and sample means (black square) are shown for firing threshold, stimulus current at threshold, and action potential latency and duration at both firing threshold and supra-threshold (+190 pA) stimulus. (**d)** Typical firing profiles of NT (black; left panels) and UT (gray; right panels) neurons, as recorded at firing threshold (+20 pA; lower panels) and in response to supra-threshold input (+190 pA; upper panels). (**e)** Neurons are classified as rapidly-adapting and slowly-adapting on the basis of the maximum number of APs fired during membrane depolarisation regardless of stimulus strength (AP Max). The majority of neurons in both NT (black) and UT (gray) groups display a rapidly-adapting profile (<7 APs). Mean AP Max for both populations shown inset. UT – untreated; NT – neurotrophin-treated; *p < 0.05 **p < *0.01*

Measures of basic electrophysiological features and action potential (AP) properties were compared across NT and UT neurons (Fig. 1). As shown in Fig. 1a, examination of the resting membrane potential (RMP) and input resistance (R_IN_) revealed no significant difference between groups. The resting membrane potential was −59.4 ± 0.6 mV in NT neurons (n = 78) compared to −59.5 ± 0.6 mV in UT neurons (n = 49), whilst R_IN_ was 637 ± 40 MOhm (n = 60) and 650 ± 68 MOhm (n = 37) respectively. However, mean membrane capacitance was 8.6 ± 0.3 pF (n = 48) for UT and 9.6 ± 0.3 pF (n = 77) for NT neurons (p = 0.020), indicating a clear difference in the size of neurons grown under the two conditions (Fig. 1a).

The assessment of AP properties included the voltage at AP onset (denoted as firing threshold), distance between stimulus onset and AP peak (denoted AP latency), and width of the AP at 50% of the overshooting component (i.e. above 0 mV; denoted as AP duration) (Fig. 1b,c). There was no difference in the firing threshold voltage for NT neurons (−44.9 ± 0.4 mV; n = 77) compared to UT neurons (−44.1 ± 0.5 mV; n = 48), nor any difference in the stimulus level required to evoke the first AP (17.3 ± 1.4 pA for NT [n = 47]; 19.8 ± 1.7 pA for UT [n = 48]). Further, there was no difference in the latency of the first AP at threshold (35.9 ± 2.1 ms for NT [n = 76]; 38.9 ± 1.6 ms for UT [n = 48]). However, AP duration (expressed by half-width as per Fig. 1b) was significantly shorter under NT conditions (963 ± 31 μs [n = 75]; compared to 1150 ± 56 μs for UT [n = 48]; p = 0.004). Supra-threshold responses (measured at +190 pA) were also slower in NT neurons (latency of 3.2 ± 0.1 ms [n = 76]) than UT neurons (2.7 ± 0.1 ms [n = 49]; p = 0.001), and this was accompanied by a significant difference in AP duration at supra-threshold levels (p < 0.001): APs of NT neurons were narrower (895 ± 26 μs [n = 76]), than those of UT neurons (1093 ± 47 μs [n = 49]). The amplitude of APs was similar in both groups (107.9 ± 1.2 mV in NT [n = 76], and 106.3 ± 1.3 mV in UT neurons [n = 49]) indicating that this did not contribute to the difference in measurement of duration.

Typical firing profiles of SGNs (Fig. 1d) show a similar response to membrane depolarisation in both NT and UT conditions. A comparison of the maximum firing rate (AP_Max_), measured as the maximum number of APs fired in response to a 300 ms supra-threshold depolarisation regardless of stimulus current (up to +190 pA), showed no difference between neurons grown under the different culture conditions. The majority of neurons fire between 1–3 APs at the onset of depolarisation (Fig. 1e). Classification of the firing profiles into either rapidly-adapting (AP_Max_ of between 1 and 6) or slowly-adapting (AP_Max_ of 7 or more) categories also indicated no difference between NT (88.3% rapidly-adapting; 11.7% slowly-adapting) and UT (87.8% rapidly-adapting; 12.2% slowly-adapting) populations. Mean AP_Max_ was 3.4 ± 0.4 in UT neurons (n = 49), compared to 3.3 ± 0.3 for NT neurons (n = 77; Fig. 1e).

### Potassium currents in the presence of neurotrophins

**Figure 2:**
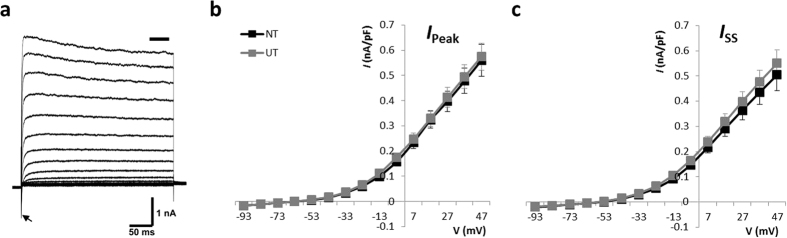
Mean current-density for outward currents evoked in response to membrane depolarisation. (**a)** The profile of a voltage-clamp recording taken from an NT neuron in response to membrane depolarisation (−93 to +47 mV) shows the typical outward current evoked in the absence of all channel blockers. The transient inward sodium current evoked at stimulus onset is indicated by an arrow. Measures were taken of the peak outward current (*I*_Peak_; (**b**) and the steady-state current (*I*_SS_; (**c)** area of measurement noted by bar in (**a**) and revealed no difference in the current density for either group. UT – untreated (n = 21); NT – neurotrophin-treated (n = 19).

Evidence that firing latency and duration differed between NT and UT groups suggested differences in potassium currents between the two populations. This followed our previous hypothesis that the change in *I*_h_ would be matched by a concurrent change in *I*_K_^6^. To determine the contribution of potassium currents, voltage-clamp recordings were examined in response to membrane depolarisation (Fig. 2a). Current-voltage relationships were obtained for measures of both peak and steady-state outward current with depolarisation of the membrane (−93 to +47 mV; 300 ms) following a long hyperpolarising prepotential (−113 mV; 500 ms). These are denoted *I*_Peak_ and *I*_SS_ respectively, and expressed as current density to account for differences in cell capacitance between groups. Overall, there was no significant difference in the outward potassium current measured for NT and UT groups (Fig. 2b,c).

**Figure 3:**
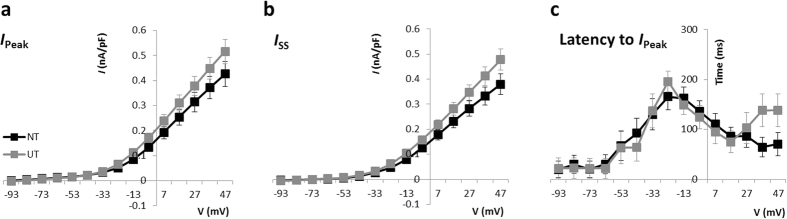
Mean current-density for outward currents following removal of sodium and hyperpolarisation-activated currents. No significant difference in current density was seen between UT and NT groups for either the peak (**a**) or steady-state (**b**) components. Assessment of the time taken between the onset of the stimulus and the peak current (**c**) revealed a similar profile for both UT and NT populations. UT – untreated (n = 19); NT – neurotrophin-treated (n = 20).

Given the larger contribution of *I*_h_ in NT neurons, these measures were also repeated in the presence of Tetrodotoxin (TTX; I μM) and 4-ethylphenylamino-1,2-dimethyl-6-methylaminopyrimidinium chloride (ZD7288; 50 μM) in order to remove any interference from the transient sodium current (*I*_Na_) and *I*_h_ respectively. Tetrodotoxin is a reversible and highly specific blocker of the voltage-gated sodium channels that underlie *I*_Na_ in SGNs, whilst ZD7288 is a selective and irreversible inhibitor of the HCN channels that mediate *I*_h_ (>97% block of *I*_h_ in SGNs^6^). As shown in Fig. 3(a,b), the amplitude of outward currents (both *I*_Peak_ and *I*_SS_) remained similar for both groups following the inclusion of these channel blockers. Moreover, the latency to peak was similar in both conditions (Fig. 3c).

### Contribution of DTX-I and PaTX-1 sensitive currents

Whilst there was little difference in the overall outward potassium current, to determine if there are differences in the relative contribution of specific potassium currents (i.e. *I*_KL_ and *I*_A_), we next examined the response to channel blockers DTX-I and PaTX-1. DTX-I, derived from black mamba venom, blocks K_V_1.1 and K_V_1.2 subunits which are linked with *I*_KL_ in SGNs. Meanwhile, *I*_A_ is linked with K_V_4.2 and K_V_4.3 subunits which are blocked by the tarantula venom, PaTX-1. Importantly, the levels of expression of both K_V_1.1 and K_V_4.2 in SGNs have been shown to be sensitive to BDNF and NT3. Each of the compounds was tested in the presence of TTX and ZD7288 to remove *I*_Na_ and *I*_h_.

**Figure 4:**
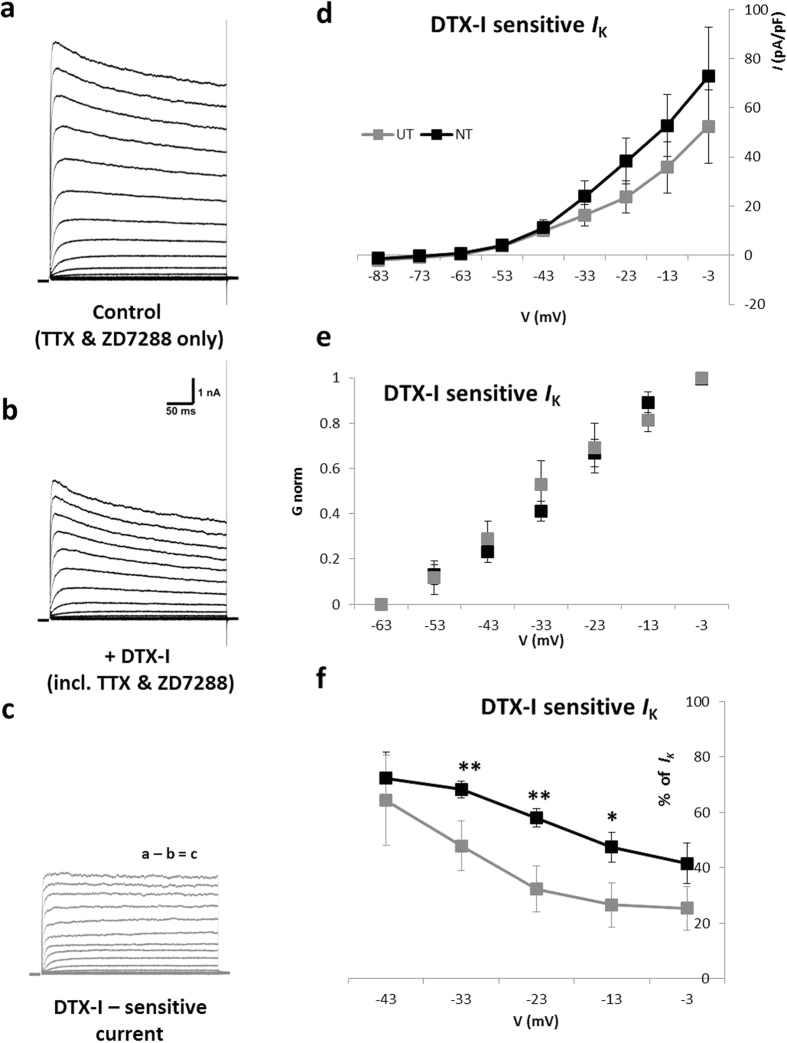
DTX-I sensitive currents in SGNs. (**a)** Typical outward currents evoked in the absence of sodium and hyperpolarisation-activated currents (example shown is an NT neuron). (**b)** A reduction in outwards currents was observed in the presence of DTX-I. (**c**) DTX-I sensitive component of the current as determined by the subtraction of recordings in (**b**) from those in (**a**). (**d)** Current-density versus voltage for the sustained portion of the DTX-I sensitive current showed no difference between UT (n = 6) and NT (n = 7) neurons below 0 mV. (**e)** Normalised conductance-voltage plots depict the activation of the DTX-I sensitive component in each condition. Conductance calculated from the steady-state DTX-I sensitive current fitted. (**f)** DTX-I sensitive current expressed as a percentage of the overall current. UT – untreated; NT – neurotrophin-treated; *p < 0.05 **p < *0.01*.

Application of DTX-I reduced the outward potassium current (Fig. 4a,b). The amplitude of the DTX-I sensitive current (Fig. 4c) did not vary over the course of the stimulation, suggesting that it underlies the non-inactivating, sustained component (*I*_SS_) of the response. The contribution of the DTX-I sensitive current (taken as the difference in current before and after drug application) was plotted as a function of membrane depolarisation (Fig. 4d), and revealed the contribution of K_V_1.1/K_V_1.2 subunits was negligible at −73 mV, and grows steadily from −53 mV. The NT group showed greater DTX-I sensitive current than UT neurons at −33 mV and above, but this was not statistically significant. Given K_V_1.1/K_V_1.2 subunits are linked with the low voltage-activated potassium current, the contribution of the DTX-I sensitive current was examined only within the voltage range at which *I*_KL_ is first activated (below 0 mV). To examine activation voltage dependence, the normalised conductance for DTX-I sensitive currents was plotted against membrane voltage (Fig. 4e). When fitted with a Boltzmann function to calculate half-maximal activation (V_h_) and slope (k) values there was no significant difference between UT (V_h_ = −29.1 ± 3.0 mV, *k* = 9.0 ± 1.5; n = 5) and NT (V_h_ = −26.9 ± 1.8 mV, *k* = 8.4 ± 0.4; n = 7) neurons. To account for individual variations in the size of the initial outward current between neurons the DTX-I sensitive current was also examined as the % change in the overall outward current (Fig. 4f). This metric revealed that in NT neurons the DTX-I sensitive component contribute 78% at −53 mV, 68% at −33 mV, decreasing to a constant 40–41% at higher voltages. The pattern of contribution was noticeably different in UT neurons. DTX-I sensitive currents contributed 87% at −53 mV, decreasing to 48% at −33 mV, and 25–30% at higher voltages. Differences between the populations were statistically significant at −33 mV (p = 0.007), −23 mV (p = 0.003) and −13 mV (p = 0.046) (Kruskal-Wallis test). These results show a difference in the contribution of DTX-I sensitive currents in the presence of neurotrophins.

**Figure 5:**
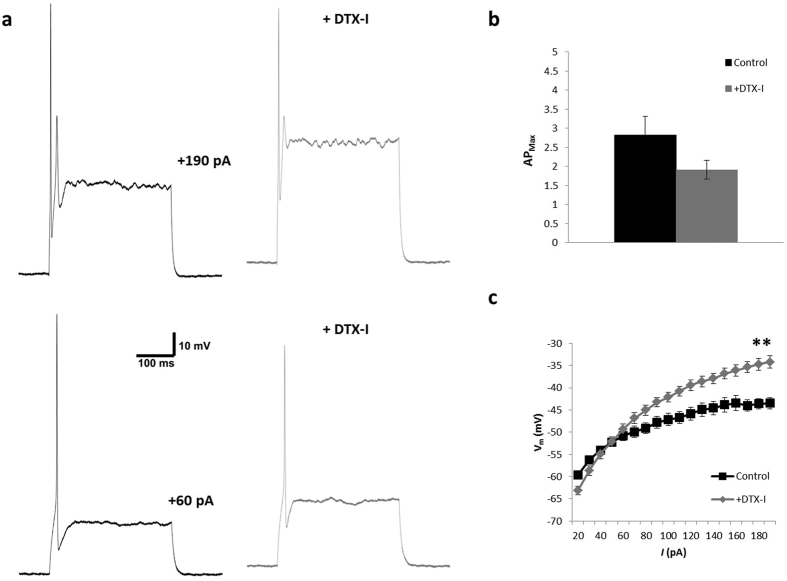
Firing frequency in the presence of DTX-I. (**a**) Action potential output did not change with the addition of DTX-I (gray; right panels) at either threshold (+60 pA; lower panels) or in response to supra-threshold input (+190 pA; upper panels). Example shown is an NT neuron. (**b**) A comparison of the maximum number of action potentials evoked (AP_Max_) before (control; black) and after application of DTX-I (gray) revealed no significant change in firing output in the presence of DTX-I (n = 11; *p* = *0.085*). However, comparison of the voltage-current relationship (measured over the last 20 ms of the 300 ms current injection) highlighted a large change in membrane voltage following application of DTX-I ((**c**) **p < *0.001*).

The contribution of K_V_1.1 and K_V_1.2 to the firing properties of SGNs was subtle in our model. Low-voltage activated potassium channels have previously been identified as key in the control of firing accommodation^37^. In this study we also monitored firing outcomes in the presence of the *I*_KL_ channel blocker DTX-I. In current-clamp, no increase in firing rate was observed in either group after application of the compound (Fig. 5a,b), nor any significant change in AP latency or duration. The only significant change observed with DTX-I was an increase in the steady-state membrane voltage in response to injected current (n = 11, p < 0.001; Fig. 5c). Data shown represent pooled results from UT (n = 5) and NT (n = 6) neurons, since outcomes were the same in both groups.

**Figure 6:**
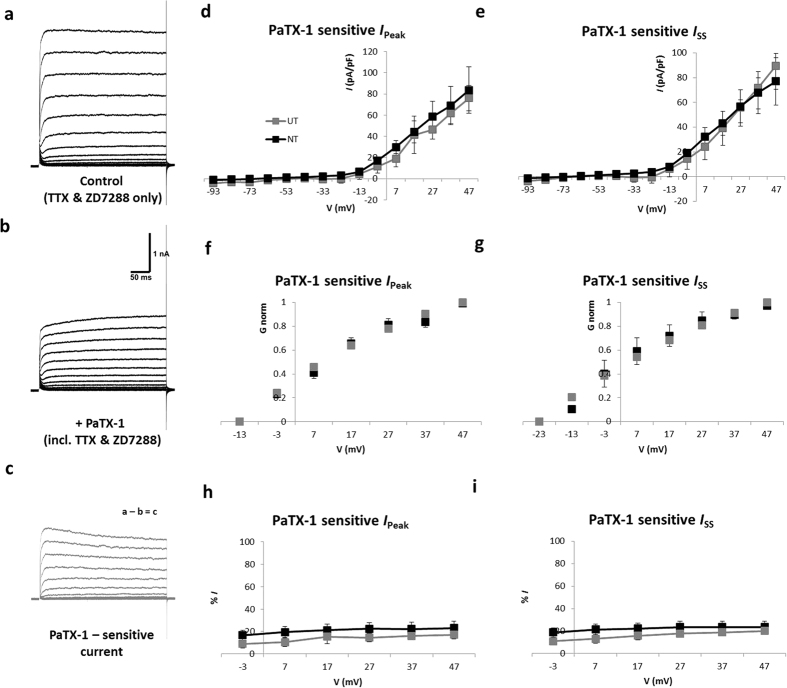
PaTX-1 sensitive currents in SGNs. (**a)** Typical outward currents evoked in the absence of sodium and hyperpolarisation-activated currents (example shown is an NT neuron). (**b)** A reduction in outward currents was observed in the presence of PaTX-I. (**c**) PaTX-I sensitive component of the current as determined by the subtraction of recordings in b from those in a. Current-density versus voltage for the peak PaTX-1 sensitive current (**d**) and sustained portion of the PaTX-1 sensitive current (**e**) showed no difference between UT (n = 4) and NT (n = 5) neurons. (**f**,**g)**. Normalised conductance-voltage plots demonstrate the activation of the PaTX-1 sensitive component of peak (*I*_Peak_) and steady-state (*I*_SS_) currents. (**h**,**i)**. PaTX-1 sensitive current expressed as a percentage of the overall current also showed no difference between groups. UT – untreated; NT – neurotrophin-treated.

The presence of PaTX-1 resulted in a reduction of outward potassium currents (Fig. 6a,b), indicating that K_V_4.2 and/or K_V_4.3 channels are present in the SGNs. The PaTX-1 sensitive current seen here displays a small peak at stimulus onset followed by a sustained component (Fig. 6c). Although an initial peak is typical of *I*_A_, this was markedly smaller than that reported by Jagger and Housley^38^. Further, whereas *I*_A_ is characterised as a transient (rapidly-inactivating) current, our PaTX-1 sensitive current showed little difference between *I*_Peak_ and *I*_SS_. Plotted as a function of membrane potential, the contribution of K_V_4.2/4.3 to both peak and steady-state currents was evident at voltages of −3 mV and above, and was present in both NT and UT neurons (Fig. 6d,e). Normalised conductance-voltage plots of the PaTX-I sensitive current were also similar for both NT and UT neurons (Fig. 6f,g). A Boltzmann function fitted to these curves revealed the half-maximal activation and slope for *I*_Peak_ (UT: V_h_ = 10.7 ± 0.7 mV, *k* = 10.6 ± 0.2; and NT: V_h_ = 11.5 ± 2.0 mV, *k* = 10.2 ± 0.7) and *I*_SS_, (UT: V_h_ = 5.6 ± 0.7 mV, *k* = 12.8 ± 0.4; and NT: V_h_ = 5.5 ± 4.7 mV, *k* = 8.8 ± 1.6) did not differ significantly. Further, no significant difference was observed in the percentage contribution of the PaTX-1 sensitive current between groups (Fig. 6h,i).

### Entrainment to pulse train stimulation

Whilst the increase in *I*_h_ that accompanies exposure to neurotrophins might be expected to alter the firing properties of SGNs, we did not see a change in firing rate when measured in response to a sustained membrane depolarisation^6^. However, given the activation range of *I*_h_ (below −73 mV), the influence of this current during sustained depolarisation may be limited. Alternatively, activation of *I*_h_ is likely greatest during pulsatile input where repolarisation can result in brief periods of membrane hyperpolarisation. Further, a key feature of SGNs is their ability to reliably follow high frequency stimulation since this is a hallmark of many acoustic stimuli, as well as the electrically encoded input from a cochlear implant.

**Figure 7:**
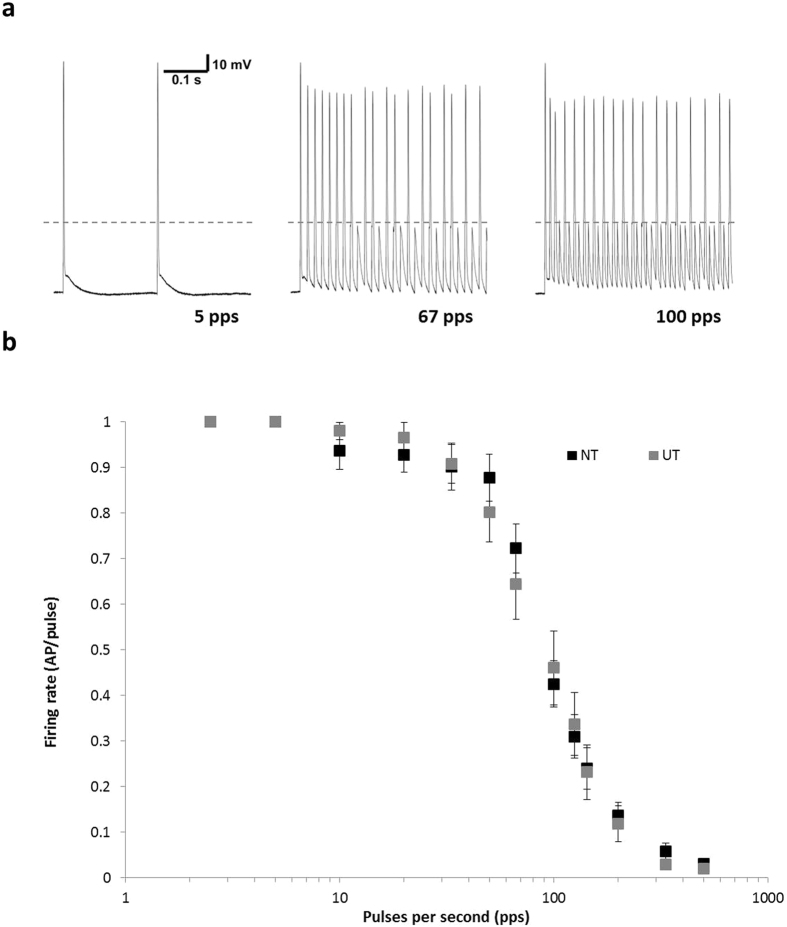
Firing entrainment during high frequency pulse trains. (**a)** Representative firing pattern of an NT neuron at 5, 67 and 100 pulses per second (pps). Dashed line denotes stimulus artefact. (**b)** Firing probability calculated as the number of action potentials (APs) fired per pulse, averaged over 50 presentations. NT neurons (black; n = 22) showed similar ability to follow pulse trains as UT neurons (gray; n = 17). Data represents mean ± S.E.M. UT – untreated; NT – neurotrophin-treated.

To determine the ability of SGNs to entrain to high frequency stimulation, firing activity was examined during a train of supra-threshold pulses (0.3 ms duration). The pulse trains were presented from 2.5 to 500 pulses per second (pps), and outcomes assessed as the probability of an AP firing in response to each pulse (averaged over a train of 50 pulses, and multiple presentations; Fig. 7). A single stimulus level was presented to each neuron, chosen as the first current above threshold at which 100% entrainment to a 2.5 pps train was obtained over multiple presentations (mean 1.80 ± 0.09 nA in NT [n = 22], and 1.53 ± 0.05 nA in UT [n = 17]). Both NT and UT neurons displayed good firing entrainment to stimuli presented between 2.5 and 33 pps, as defined by a firing probability greater than 0.9. At 50 pps, the probability decreased slightly to 0.88 for NT, and 0.80 for UT. Whilst the difference between firing entrainment in NT and UT populations was most marked at 67 pps, this did not represent a significant change: firing entrainment was 0.72 for NT neurons, compared to only 0.64 for UT neurons. Firing probability fell below 0.5 for both groups in response to pulse trains presented at 100 pps and above. Stimulation at 500 pps produced only a single AP at stimulus onset in most neurons, regardless of treatment conditions. A further comparison was made between populations using only responses to stimuli presented at 1.5 nA (n = 10 for NT; n = 13 for UT), and also showed no difference between firing entrainment with treatment (data not shown). These results indicate that the changes in *I*_h_ and DTX-I-sensitive currents evoked in response to BDNF and NT3 are insufficient to alter firing patterns during high frequency stimulation.

## Discussion

This study provides the first description of potassium currents in SGNs following exposure to both BDNF and NT3, and the response of neurotrophin-treated neurons to high frequency stimulation. The findings reported herein demonstrate that combined neurotrophin delivery increases firing latency and action potential duration and induces a small change in the contribution of DTX-I-sensitive currents. However, there was no change in the total outward potassium current, nor any impact on firing adaptation or entrainment during high frequency pulse trains. This supports our previous assertion that combined BDNF and NT3 exposure maintains the inherent firing pattern of SGNs, and that this is the preferred response from a therapeutic perspective.

The recordings presented here demonstrate that both PaTX-1 (K_V_4.2/K_V_4.3) and DTX-I (K_V_1.1/1.2) sensitive currents are present in dissociated rat SGNs *in vitro*. Whilst DTX-sensitive currents have previously been reported in SGNs^37^,^39^,^40^, this study provides the first description of a PaTX-1-sensitive current in primary auditory neurons. PaTX-1 blocks K_V_4.2 and K_V_4.3, channels which have been linked with *I*_A_, a current previously observed in rat SGNs *in situ*^38^. The PaTX-1 sensitive current observed in this study appears to underlie the initial peak at stimulus onset, as well as contributing to the sustained response (*I*_SS_) at higher voltages. Although this differs somewhat from the properties of *I*_A_ reported by Jagger and Housley^38^ – a fast-onset, transient (rapidly-inactivating) current – it nonetheless demonstrates that K_V_4.2/K_V_4.3 mediated currents contribute around 10–20% of *I*_Peak_ and *I*_SS_.

Previous studies indicate that expression of both K_V_4.2 and K_V_1.1 are sensitive to NT3 and BDNF^2^. Expression of K_V_4.2 increased in the presence of NT3, whilst application of BDNF increased its expression in basal neurons only. Alternatively, K_V_1.1 increased in response to BDNF, but was unchanged by NT3. An increase in *I*_KL_ and/or *I*_A_ has also been reported to accompany increases in *I*_h_ in central auditory neurons^31^,^34^ and lobster stomatogastric neurons^32^. Based on these outcomes, we hypothesized that the currents mediated by these channels would increase in the presence of both BDNF and NT3. Instead, our results show that the amplitude of both K_V_4.2/K_V_4.3 and K_V_1.1/1.2 mediated currents are similar in both NT and UT culture conditions. The overall outward current was also consistent between the two groups. Nonetheless, the DTX-I sensitive current controlled by K_V_1.1 and K_V_1.2 channels played a larger role in *I*_KL_ in neurons exposed to neurotrophins. It remains to be determined whether this represents a change in the relative contribution of another low voltage-activated channel.

It is also interesting to note that the firing rate did not change with addition of DTX-I. A number of earlier studies have reported a significant increase in firing output following application of dendrotoxin, increasing the firing rate of both rapidly-adapting and slowly-adapting neurons in its presence^37^,^39^,^41^,^42^. That result implicated K_V_1.1 and K_V_1.2 in the control of firing frequency. The absence of such effects in our experiments indicates that K_V_1.1 and K_V_1.2 are not the main drivers of firing adaptation in our population of SGNs. This result mirrors that previously described by Szabo and colleagues^40^ in which DTX-sensitive currents were recorded from dissociated adult guinea pig SGNs, but firing output was unaffected by application of the toxin. Although enzymatically dissociated SGN cultures were employed by both studies, this method was also used in two separate mouse studies^39^,^42^. Using dissociated mouse SGNs from postnatal day 10–12^39^ or 12–15^42^ animals cultured for 1–3 days *in vitro*, these studies both reported similar findings to those of Mo^37^ and Liu^41^, using postnatal day 3–8 mouse SGN explants cultured for up to 2 weeks *in vitro*. This suggests that species differences are a possible cause of differences in adaptation outcomes, rather than age of the neurons, length of time in culture, or exposure to enzymatic dissociation.

In contrast to our earlier investigation^6^, the current study found an increase in latency (to supra-threshold stimulation) and decrease in AP width (for both threshold and supra-threshold responses) with exposure to neurotrophins. Whilst the larger sample size presented here may in part account for the emergence of such changes, it might also highlight differences related to neurotrophin concentration. The present study examined exposure to BDNF and NT3 at the more physiologically-relevant level of 10 ng/ml each, compared to the 50 ng/ml employed previously^6^. Although hyperpolarisation-activated currents are of similar amplitude using either concentration^6^, changes in firing latency and duration are only evident with BDNF and NT3 at 10 ng/ml. Indeed, a concentration dependent alteration in firing properties has previously been reported for NT3^7^, and found firing latency increased most markedly in the presence of 10 ng/ml NT3. Other studies have also reported an increase in firing latency with NT3, and a decrease in latency following exposure to BDNF (at 5 ng/ml^2^). Whilst use of combined NT3 and BDNF might be expected to offset the influence of either neurotrophin when presented on their own, the present data suggests that NT3 is more influential in this regard when both neurotrophins are included in the culture. Although the control of latency has been attributed to K_V_1.1, with an increase in K_V_1.1 linked to a decrease in latency, this seems unlikely in the present model. Our results suggest that the contribution of K_V_1.1/K_V_1.2 channel subunits increases with combined neurotrophins in concert with an increase in latency. Further, application of DTX-I had no effect on firing latency.

Despite the neurotrophin-mediated changes observed (AP latency and duration, *I*_h_ and the contribution of DTX-I sensitive currents), the overall maintenance of firing adaptation and firing frequency suggests that exposure to a combination of BDNF and NT3 has little or no effect on global neuronal activity. Thus, whilst BDNF and NT3 alone can propagate opposing activity phenotypes^2^,^26^, this study confirms earlier reports that the presence of both neurotrophins preserves firing behaviour^6^,^26^. This may be advantageous in a clinical setting, where the end goal is to maintain a healthy and functional population of auditory neurons for stimulation via the cochlear implant.

## Materials and Methods

### Ethics statement

This study was carried out in accordance with the Code of Practice for the Care and Use of Animals for Scientific Purposes of the National Health and Medical Research Council of Australia. All protocols were approved by the Animal Research and Ethics Committee of the Royal Victorian Eye and Ear Hospital and the Bionics Institute, Melbourne, Australia.

### Culture methods

Cultures of primary auditory neurons were prepared from the cochlea of post-natal day 4 to 7 (P4–P7) Wistar rat pups as described previously^6^. Briefly, the modioli of up to 10 rat pups were isolated and placed into chilled Neurobasal media (NBM) comprising: Neurobasal A (Invitrogen), N2 and B27 supplements (Invitrogen), L-glutamine (Invitrogen), and Penicillin/Streptomycin (Invitrogen). The tissue was dissociated via both enzymatic digestion (37 ^o^C for 10 mins) and gentle trituration. Dissociated cells were plated onto circular glass coverslips (10 mm diameter; Menzel-Glaser) pre-coated with poly-ornithine (500 μg/ml; Sigma) and mouse laminin (0.01 mg/ml; Invitrogen). Cells were cultured in NBM only (untreated) or NBM supplemented with human recombinant BDNF and NT3 (each at 10 ng/ml; Peprotech). Cultures were maintained at 37 ^o^C (10% CO_2_). NBM was replaced at 4 hrs, then replenished daily.

### *In vitro* electrophysiology

Coverslip were transferred to the recording chamber of the microscope (AxioExaminer D1, Carl Zeiss Pty Ltd, Germany) fitted with a 40x water-immersion objective lens. Cells were continuously perfused with physiological saline of the following composition (in mM): 137 NaCl, 5 KCl, 10 HEPES, 1 MgCl_2_, 2 CaCl_2_, 10 glucose (pH 7.35; 300–305 mOsmol/kg). Neurons were visualised with Dodt optics, and a monochrome CCD camera (Spot RT SE18, Diagnostic Instruments) and chosen for recordings on the basis of the following features: phase-bright, round-to-oval soma (~15–20 μm diameter), and prominent eccentric nucleus with single nucleolus. Bipolar processes were often identified in neurons displaying these features. Whole-cell patch-clamp recordings were made at room temperature using borosilicate microelectrodes (tip resistance 2–8 MΩ; 1.0 mm O.D., 0.58 mm I.D.) filled with solution containing (in mM): 115 K-gluconate, 10 HEPES, 7 KCl, 0.05 EGTA, 2 Na_2_ATP, 2 MgATP, 0.5 Na_2_GTP (pH 7.3; 294 mOsmol/kg). All chemicals were purchased from Sigma-Aldrich (Sydney, Australia) unless otherwise indicated. Tetrodotoxin (TTX; Alomone, Jerusalem, Israel), 4-ethylphenylamino-1,2-dimethyl-6-methylaminopyrimidinium chloride (ZD7288; Tocris Bioscience, Bristol, UK), Dendrotoxin-I (DTX-I; Alomone), and Phrixotoxin-1 (PaTX-1; Alomone) were diluted daily to final concentrations in the bath perfusate, and administered by superfusion via a gravity-fed system. Final concentrations used were: 100 nM DTX-I, 100 nM PaTX-1, 1 μM TTX, 50 μM ZD7288.

Signals were recorded with a MultiClamp 700B amplifier (Molecular Devices, Sunnyvale, CA, USA), and data acquisition system (Digidata 1440A, Molecular Devices), and AxoGraph X analysis software (AxoGraph Scientific, Sydney, Australia). Records were digitized at 50 kHz and filtered at 10 kHz. Series resistance (*R*_S_) was monitored in response to 10 mV voltage step (mean 14.0 ± 0.3 MΩ; recordings in which resistance exceeded 20 mΩ were excluded), and partially compensated online (70%). No corrections were made for voltage errors for uncompensated *R*_S_. In current clamp, bridge balance and pipette capacitance neutralization were applied to compensate for errors due to *R*_S_. All recordings were made from a holding potential of −73 mV. Corrections for liquid junction potential were made offline.

All recordings were made at 2 days *in vitro* to limit the impact of space constants that occur with increased neurite length following extended periods in culture. Neurotrophin-mediated changes in hyperpolarisation-activated currents (*I*_h_) were previously identified between untreated and neurotrophin-treated neurons at this time point^6^. All cells included for analysis exhibited the ability to fire action potentials (overshooting action potential), and where recordings were made in voltage-clamp, evidence of a fast inward sodium current was always observed. Cultures contained neurons from multiple (average of 8) animals, and represented all regions of the cochlea (apical, middle and basal).

### Data analysis

Membrane capacitance and input resistance were calculated from the response to a +10 mV test pulse, as a function of current decay or steady-state respectively. Firing properties were examined in response to a 300 ms depolarizing current pulse (10 pA to +190 pA; +10 pA steps). The maximum firing rate (AP_Max_) was defined as the maximum number of APs fired in response to a single 300 ms supra-threshold depolarization regardless of stimulus current. Using the classification criteria established by Mo, Adamson and colleagues^36^,^43^, firing accommodation was identified as slowly-adapting where AP_Max_ was 7 or greater and rapidly-adapting where 1 to 6 APs were recorded. Voltage-dependence of activation was determined by fitting the normalised conductance-voltage relationship for steady-state currents with a Boltzmann function, G − G_min_/G_max_ − G_min_ = 1/(1 + exp ((V_h_ − V)/*k*)) where V is membrane potential, V_h_ is the half-maximal activation and *k* is the slope. Statistical significance determined using Kruskal-Wallis or Student t-tests (MiniTab 17); data were considered significant where p < 0.05. Results presented as mean ± SEM; n being the number of neurons in which measurements were made.

## Additional Information

**How to cite this article**: Wright, T. *et al*. Firing frequency and entrainment maintained in primary auditory neurons in the presence of combined BDNF and NT3. *Sci. Rep.* **6**, 28584; doi: 10.1038/srep28584 (2016).
